# Associations between Nitric Oxide Synthase Genes and Exhaled NO-Related Phenotypes according to Asthma Status

**DOI:** 10.1371/journal.pone.0036672

**Published:** 2012-05-09

**Authors:** Emmanuelle Bouzigon, Florent Monier, Mekki Boussaha, Nicole Le Moual, Hélène Huyvaert, Régis Matran, Sébastien Letort, Jean Bousquet, Isabelle Pin, Mark Lathrop, Francine Kauffmann, Florence Demenais, Rachel Nadif, on behalf of the EGEA Cooperative Group

**Affiliations:** 1 Inserm, U946, Paris, France; 2 Université Paris Diderot, Sorbonne Paris Cité, Institut Universitaire d'Hématologie, Paris, France; 3 Fondation Jean Dausset-Centre d'Etude du Polymorphisme Humain (CEPH), Paris, France; 4 Inserm, U1018, CESP Centre for Research in Epidemiology and Population Health, Respiratory and Environmental Epidemiology Team, Villejuif, France; 5 Université Paris-Sud 11, UMRS 1018, Villejuif, France; 6 Université Lille Nord de France, Lille, France; 7 CHU, Lille, France; 8 CHU Arnaud de Villeneuve, Montpellier, France; 9 Inserm, U823, Epidémiologie environnementale appliquée à la reproduction et la santé respiratoire, Institut Albert Bonniot, Grenoble, France; 10 Université Joseph Fourier, Grenoble, France; 11 Pédiatrie, CHU de Grenoble, Grenoble, France; 12 Commissariat à l'Energie Atomique, Centre National de Génotypage, Evry, France; Louisiana State University Health Sciences Center, United States of America

## Abstract

**Background:**

The nitric oxide (NO) pathway is involved in asthma, and eosinophils participate in the regulation of the NO pool in pulmonary tissues. We investigated associations between single nucleotide polymorphisms (SNPs) of NO synthase genes (*NOS)* and biological NO-related phenotypes measured in two compartments (exhaled breath condensate and plasma) and blood eosinophil counts.

**Methodology:**

SNPs (N = 121) belonging to *NOS1, NOS2* and *NOS3* genes were genotyped in 1277 adults from the French Epidemiological study on the Genetics and Environment of Asthma (EGEA). Association analyses were conducted on four quantitative phenotypes: the exhaled fraction of NO (Fe_NO_), plasma and exhaled breath condensate (EBC) nitrite-nitrate levels (NO2–NO3) and blood eosinophils in asthmatics and non-asthmatics separately. Genetic heterogeneity of these phenotypes between asthmatics and non-asthmatics was also investigated.

**Principal Findings:**

In non-asthmatics, after correction for multiple comparisons, we found significant associations of Fe_NO_ levels with three SNPs in *NOS3* and *NOS2* (P≤0.002), and of EBC NO2–NO3 level with *NOS2* (P = 0.002). In asthmatics, a single significant association was detected between Fe_NO_ levels and one SNP in *NOS3* (P = 0.004). Moreover, there was significant heterogeneity of *NOS3* SNP effect on Fe_NO_ between asthmatics and non-asthmatics (P = 0.0002 to 0.005). No significant association was found between any SNP and NO2–NO3 plasma levels or blood eosinophil counts.

**Conclusions:**

Variants in NO synthase genes influence Fe_NO_ and EBC NO2–NO3 levels in adults. These genetic determinants differ according to asthma status. Significant associations were only detected for exhaled phenotypes, highlighting the critical relevance to have access to specific phenotypes measured in relevant biological fluid.

## Introduction

The endogenous nitric oxide (NO) plays a key role in physiological regulation of airway functions and is implicated in airway diseases such as asthma [1], [2]. In biological fluids, the half-life of NO is extremely short due to its rapid oxidation to nitrite (NO_2_-) and nitrate (NO_3_-) [3]. NO_2_- and NO_3_- should now be viewed as storage pools for NO-like bioactivity, thereby complementing the NO synthase (NOS)-dependent pathway [3].

**Table 1:** Characteristics of non-asthmatics and asthmatic subjects.

	Non-asthmatic Subjects N = 783	Asthmaticsubjects N = 494
Age, year, mean ± SD	46.6±15.8	39.8±16.4
Sex, women, %	53.9	49.0
Smoking habits:		
Never smokers, %	51.1	49.0
Ex-smokers, %	27.8	25.5
Current smokers, %	21.1	25.5
**Asthma**		
FEV_1_ % predicted, mean ± SD	106.7±16.5	95.4±19.0
Methacholine test, PD20≤4 mg,* n (%)	493 (27.4)	285 (70.5)
Inhaled corticosteroids, last 3 months, %	0.0	17.6
Inhaled corticosteroids, last 12 months, %	3.5	48.4
Age at onset of asthma, n:	/	*437*
≤4 yrs, %	NA	30.4
]4−16] yrs, %	NA	34.3
>16 yrs, %	NA	35.2
Asthma severity according to GINA 2006 guidelines, n:		*469*
Intermittent, %	NA	55.4
Mild persistent, %	NA	3.8
Moderate persistent, %	NA	12.3
Severe persistent, %	NA	28.4
**SPT+ and allergic rhinitis**		
SPT+#, %	39.1	80.6
SPTQ, median [Q1–Q3$]	0 [0–1]	2 [1]–[4]
IgE, IU/ml, median [Q1–Q3]	43.3 [16.5–122]	162 [64.7–377]
Allergic rhinitis, %	22.1	60.1
**Biological phenotypes, median [Q1-Q3]**		
Eosinophils/mm^3^	130 [100–200]	200 [140–320]
FeNO, ppb	14.9 [9.05–19.5]	18.0 [11.9–28.9]
Plasma NO_2_–NO_3_, µM	35.7 [24.8–51.6]	36.5 [25.9–50.1]
EBC NO_2_–NO_3_, µmol/mg	2.01 [0.92–3.78]	1.90 [0.85–4.06]

# Skin Prick Test positivity (SPT+) was defined by a mean wheal diameter≥3 mm than the negative control for at least one of 11 aeroallergens. SPTQ: number of positive test.

IgE: immunoglobulin E, NA: not available. *Not performed if FEV_1_<80% predicted.

$ Q1–Q3 = first and third quartile.

The measure of exhaled fraction of NO (Fe_NO_) is considered as a marker for eosinophilic inflammation in asthma, and can be used together with sputum eosinophil counts to titrate anti-inflammatory treatment in asthmatic patients [4]–[6]. There are *in vitro* evidences that human blood eosinophils produce NO and participate in the regulation of the NO pool in pulmonary tissues [7], [8]. The NO modulates the Th1/Th2 balance by favoring Th2 response and IL-5 production and thus recruiting eosinophils into the airways [7], [8]. Moreover, inactivation of the inducible Nitric Oxide Synthase (iNOS), one of the key enzymes in the formation of NO, decreases the eosinophil count in bronchial alveolar lavage and in blood [9].

Nitric oxide is endogenously produced by three nitric oxide synthase isoforms (NOSs, EC 1.14.13.39): a neuronal isoform (nNOS or NOS-1), an inducible isoform (iNOS or NOS-2) and a vascular endothelial isoform (eNOS or NOS-3) [10]. These proteins are encoded by three distinct genes: *NOS1*, *NOS2* (previously *NOS2A*) and *NOS3* located on chromosome 12, 17 and 7 respectively. The three NOS isoforms are expressed by human airway epithelial cells [11]. It is admitted that NOS-1 and NOS-3 control low levels of NO to perform physiological functions whereas NOS-2 shows increased expression during inflammation and is expressed predominantly in T cells, macrophages and epithelial cells [12]. Further, NOS-2 activity is the major determinant of nitric oxide levels in exhaled breath [13].

Despite the biological evidence of a physiological link between eosinophils and NO, only one study has investigated associations between variants in *NOS* genes and both eosinophils and NO [14]. The authors reported associations between allele 3 of the intron 4 (GT)n repeat belonging to *NOS2* and both percentage of blood eosinophils and serum nitric oxide levels in 230 families ascertained through asthma. However, only four variants in *NOS2* gene were investigated in that study, and the modifying effect of asthma was not studied.

The main objective of the present study was to investigate associations between 121 Single Nucleotide Polymorphisms (SNPs) of *NOS1*, *NOS2* and *NOS3* genes with 1) the three biological phenotypes of the nitrate-nitrite-NO pathway [3]: Fe_NO_ levels, total nitrite-nitrate levels measured both in plasma and in exhaled breath condensate, and 2) blood eosinophil counts, in 1277 asthmatic and non-asthmatic adults from the French Epidemiological study on the Genetics and Environment of Asthma (EGEA). Moreover, we tested for heterogeneity of genetic variant effect on these four phenotypes between non-asthmatic and asthmatic subjects. In this study, we had the unique possibility to compare the associations between *NOS* variants and total nitrite-nitrate levels measured in two compartments: plasma and exhaled breath condensate.

## Results

Characteristics of non-asthmatic and asthmatic subjects are shown in Table 1. Subjects with current asthma were younger than non-asthmatic subjects, had significantly lower % predicted FEV_1_, more often hyperresponsiveness, and were more often atopic (all P<0.0001). Eosinophil counts (EOS) and Fe_NO_ levels were significantly higher in asthmatic subjects than in non-asthmatic subjects (P<0.0001). No significant difference was observed for plasma or EBC NO_2_–NO_3_ levels between both groups.

Table 2 presents pair-wise associations between the three biological phenotypes of nitrite-nitrate-NO pathway and eosinophil counts. There was no association between plasma and EBC NO_2_–NO_3_ levels both in non-asthmatic and asthmatic subjects. Fe_NO_ levels were positively associated with blood EOS counts both in non-asthmatic and asthmatic subjects. In non-asthmatic subjects, plasma NO_2_–NO_3_ was positively associated with blood EOS counts, and both plasma and EBC NO_2_–NO_3_ levels were positively associated with Fe_NO_ levels. In asthmatic subjects, EBC NO_2_–NO_3_ was positively associated with EOS counts. Among asthmatic subjects, associations remained similar when analyses were restricted to those who did not receive inhaled corticosteroids (ICS) in the last three months.

### Single marker analysis

In non-asthmatic subjects, two SNPs in *NOS3* (rs2853796 and rs1549758, P≤0.0009, LD: D′ = 1 and r^2^ = 0.08) and one SNP in *NOS2* (rs6505510, P = 0.002) were significantly associated with Fe_NO_ levels after adjustment for multiple comparisons (Table 3 and Table S2). The CC genotype of rs6505510, C and T alleles of rs1549758 and rs2853796 respectively were associated with increased levels of Fe_NO_. We observed significant associations between EBC NO_2_–NO_3_ levels and one SNP belonging to *NOS2* (rs4795067, P = 0.002), subjects with at least one copy of G allele having higher NO_2_–NO_3_ levels (Table 3 and Table S3). No significant association was observed between any SNP and plasma or blood phenotypes (NO_2_–NO_3_ plasma levels and EOS count (Tables S4 and S5).

In asthmatic subjects, after adjustment for multiple comparisons, a single significant association was detected between Fe_NO_ levels and one SNP in *NOS3* (rs743507, P = 0.004), the C allele of rs743507 being associated with increased levels of Fe_NO_ (Table 3 and Table S2). We did not observed any association signal between SNPs and the three other phenotypes under study that reached the critical p-value thresholds taking into account multiple testing.

There was significant heterogeneity of *NOS3* SNP effect on Fe_NO_ levels according to asthma status (Table S2), with opposite allele effects between subjects with and without asthma. The most significant interaction was observed for rs743507 (P_Int_ = 0.0002).

**Table 2:** Pair-wise association of eosinophil count and NO-related phenotypes in non-asthmatic and asthmatic subjects.

		Eosinophils/mm^3^*				FeNO, ppb#				Plasma NO_2_–NO_3_, µM*		
	n	Estimate	SD	p-value	n	Estimate	SD	p-value	n	Estimate	SD	p-value
**Non-asthmatic subjects**												
EBC NO_2_-NO_3_,mol/mg*	534	−0.03	0.07	0.67	262	**0.27**	0.11	0.02	523	0.002	0.08	0.97
Plasma NO_2_–NO_3_, µM*	661	**0.11**	0.04	0.001	327	**0.13**	0.05	0.008				
FeNO, ppb#	364	**0.20**	0.04	<0.0001								
**Asthmatic subjects**												
EBC NO_2_-NO_3_,mol/mg*	346	**0.21**	0.1	0.03	202	0.14	0.12	0.24	336	0.15	0.12	0.22
Plasma NO_2_–NO_3_, µM*	418	0.03	0.04	0.40	240	0.07	0.05	0.12				
FeNO, ppb#	257	**0.32**	0.06	<0.0001								

Estimates are adjusted for *age and sex, or ^#^age, sex, height, smoking and centre (GEE regression methods).

### Multi-marker analysis

In non-asthmatic subjects, stepwise regression analyses showed independent effect of two *NOS2* SNPs: rs12601458 and rs6505510 (P = 0.01 and P = 0.003 respectively, with D′ = 0.38 and r^2^ = 0.02) and a single *NOS3* SNP (rs2853796, p = 0.0007) on Fe_NO_ levels (Table 4).

Multiple regressions performed in non-asthmatic subjects showed independent effects of two *NOS2* SNPs: rs4795067 and rs4796190 (P = 0.002 and P = 0.01 respectively, with D′ = 0.09 and r^2^ = 0.002) on EBC NO_2_–NO_3_ levels (Table 4).

Among asthmatics, a single *NOS3* SNP (rs743507) was associated with FeNO levels with similar results in the multi-marker analysis as compared to the single marker analysis (n = 189, estimate = 0.0745, 95%CI (0.017,0.132), P = 0.01).

**Table 3:** Associations between SNPs belonging to *NOS2* and *NOS3* and two quantitative phenotypes measured in the exhaled breath condensate (Fe_NO_ level and NO_2_–NO_3_ level) in non-asthmatic and asthmatic subjects–Single marker analysis.

								Exhaled phenotypes					
								FeNO, ppb			NO2-NO3, µmol/mg		
Chromosome	Gene	Genotyping method	Marker	Position (bp)	Major allele*	Minor allele	MAF†	beta	95% CI #	P	beta	95% CI #	P
Non-asthmatic subjects													
17	***NOS2*** ‡	610K	rs4795067	23130802	A	G	0.35	−0.04	−0.10; 0.03	0.26	0.13	0.04; 0.22	**0.002**
		610K	rs6505510	23203917	A	C	0.40	0.10	0.04; 0.17	**0.002**	0.08	−0.01; 0.17	0.07
7	***NOS3*** ‡	Taqman	rs1549758	150326659	C	T	0.35	−0.06	−0.10; −0.02	**0.0009**	0.003	−0.06; 0.06	0.92
		Taqman	rs2853796	150334848	G	T	0.48	0.06	0.02; 0.09	**0.0007**	−0.02	−0.08; 0.04	0.46
Asthmatic subjects													
7	***NOS3*** ‡	610K	rs743507	150338421	T	C	0.27	0.08	0.03; 0.14	**0.004**	0.02	−0.07; 0.11	0.65

**In bold P-values surviving the adjustment** (P≤0.0024 or P≤0.01 for *NOS2* and *NOS3* respectively).

* Major allele was consider as baseline allele

† MAF: Minor allele frequency

# CI: confidence interval

‡ Analyses were conducted under an additive genetic model for *NOS3* SNPs and under recessive or dominant genetic models for *NOS2* SNPs and FeNO and EBC NO_2_–NO_3_ levels respectively.

## Discussion

The present study investigated for the first time simultaneously the associations between the three genes of the *NOS* family and biological phenotypes in the nitrate-nitrite-NO pathway measured in plasma and in exhaled breath condensate (EBC), and blood eosinophil count. The compartmentalized formation of NO (plasma and exhaled breath condensate) and the modifying effect of asthma status on these associations were also investigated. Regardless of asthma status, significant associations were found only for exhaled biological phenotypes (Fe_NO_ and EBC NO_2_–NO_3_ levels). The association of Fe_NO_ with *NOS3* variants clearly differed between asthmatics and non-asthmatics.

In the present study, we only observed significant associations between *NOS* polymorphisms and the two exhaled phenotypes (Fe_NO_ and EBC NO_2_–NO_3_) whereas biological phenotypes under study, especially NO_2_–NO_3_, were measured both in blood and in exhaled breath condensate. Whatever asthma status, Fe_NO_ levels were associated with genetic variants belonging to *NOS3*. In non-asthmatic subjects, both Fe_NO_ and EBC NO_2_–NO_3_ levels were associated with genetic variants belonging to *NOS2* which encodes NOS-2, the major enzyme producing NO in exhaled breath [13]. No association was detected for plasma NO_2_–NO_3_ levels neither in non-asthmatic nor in asthmatic subjects. Moreover, plasma NO_2_–NO_3_ levels were not correlated to EBC NO_2_–NO_3_ levels in our study. Production of NO_2_–NO_3_ in EBC differs from that in plasma due to their compartmentalization: ionized nitrate and nitrite (not volatile) may arise in EBC from NO after reaction with oxygen [15] or from activated immune cells presents in the lining of the lungs [16]. Opposite to EBC, the production of NO_2_–NO_3_ in plasma is more complex where it derives from endogenous as well as dietary sources [17]. A NOS independent formation of NO also occurs in mammal blood and tissues through several enzymatic and nonenzymatic routes [18]. Overall, the specificities of NO metabolism in plasma and EBC may partly explain our results. They are consistent with the hypotheses of Villanueva and Giulvini [19], for whom such compartmentalized production of NO better explain its different functions and roles in pathophysiology, and highlight the interest to carefully choose the fluid in which a biological phenotype should be measured to study their association with genetic factors.

**Table 4:** Associations between SNPs belonging to *NOS2* and *NOS3* with Fe_NO_ and total NO_2_–NO_3_ levels in exhaled breath condensate in non-asthmatic subjects – Multivariate analysis.

					Non-asthmatic subjects		
Gene	Marker	Baseline allele or genotype *versus* Risk allele or genotype	Risk allele/genotype frequency	LD D'/r^2^	Estimate	95%CI‡	p-value
	**FeNO, ppb** * **(N = 341)**						
*NOS2*							
	rs12601458	CC+CA vs AA	0.14	0.38/0.02	−0.216	−0.382; −0.050	0.01
	rs6505510	AA+AC vs CC	0.40		0.097	0.034; 0.159	0.003
	**FeNO, ppb** * **(N = 311)**						
*NOS3*	rs2853796	G vs T	0.48		0.056	0.024; 0.088	0.0007
	**EBC NO_2_-NO_3_, µmol/mg** † **(N = 490)**						
*NOS2*	rs4795067	AA vs AG+GG	0.35	0.09/0.002	0.137	0.051; 0.223	0.002
	rs4796190	TT vs TC+CC	0.33		0.103	0.020; 0.186	0.015

* Adjusted for age, sex, height, smoking, centre and principal components.

† Adjusted for age, sex and principal components.

‡ 95% confidence interval of regression coefficient.

Inhaled corticosteroid (ICS) can modify FeNO levels, but no association between ICS and NO_2_–NO_3_ in EBC has yet been evidenced [20], [21]. To avoid any masking effect of ICS on the association between genetic determinants and FeNO levels, we restricted our analyses in subjects with asthma to those who did not received ICS within the three months preceding the measurement of exhaled NO levels. A relatively small proportion (17%) of subjects with current asthma did take ICS in that period and such ICS use did not significantly modify the association between FeNO and asthma (data not shown). The significant associations detected when restricting the analysis to subjects with asthma who did not receive ICS in the last three months before the FE_NO_ measurement were stronger.

To date, very few studies have investigated associations between Fe_NO_ level and polymorphisms in the three *NOS* genes in adults with asthma [22], [23], [24], or simultaneously considered blood eosinophil count and serum nitric oxide level [14]. Three of these studies have studied very few polymorphisms whithin each gene (one to four variants) and no study has sought for association with nitrite-nitrate levels. The first two studies conducted in less than 100 asthmatic subjects, reported association between Fe_NO_ levels and genetic variants in *NOS3* (G894T) [22] and *NOS1* (AAT repeats in intron 13 (formely intron 20) [23]). One study investigated four microsatellites belonging to *NOS2* in families with asthma and reported association between allele 3 of the intron 4 (GT)n repeat and both percentage of blood eosinophils and serum nitric oxide level [14]. In the present study, a single association between Fe_NO_ levels and *NOS3* variants was detected in asthmatics (rs743507, P = 0.004 with P_Int_ = 0.0002). Methodological differences as correction for multiple comparisons and others related to the population studied (sample size, asthma definition, measurements of biological phenotypes) may partly explain the discrepancy between all these results. Several hypotheses may explain that overall we found a reduced number of significant signals among subjects with asthma than in those without asthma: the interplay of complex mechanisms including genetic and/or environmental factors and their interactions, some heterogeneity among the subjects with asthma in our population, which included more than 50% with intermittent asthma, or a lack of power due to the small sample size of subjects with asthma. Indeed the lack of internal replication within our study between the two groups might also be due to chance findings among those without asthma. Such a possibility was addressed within our analysis by careful consideration of multiple testing. Further, recent results in an independent population support our findings and confirm significant heterogeneity between subjects with and without asthma [24]. In that study conducted in 1700 Swedish adults, Dahgam *et al.* [24] reported significant association between rs7830 belonging to *NOS3* and FeNO levels only in subjects with asthma. Interestingly, this SNP is in linkage disequilibrium with rs743507 (D′ = 1, r^2^ = 0.2) for which we detected significant association with FeNO level in subjects with asthma only, and the T risk allele of rs7830 is associated with the T risk allele of rs743507. The region of strong LD where these two SNPs are located is common to two genes *NOS3* and *ATG9B* (ATG9 autophagy related 9 homolog B) which functionally interact: *ATG9B* gene functions as the antisense to and in the posttranscriptional regulation of endothelial nitric oxide synthase 3.

Our results extend observations on the genetic determinants of FeNO levels conducted in children, more often studied than adults until now. In children without asthma [25], significant associations have been evidenced between FeNO levels and genetic variants of *NOS2* promoter region (including rs1889022 and rs10853181, two SNPs in LD with rs6505510 (D′ = 1, r^2^ = 0.4) associated with FeNO levels in adults without asthma in our study). The C allele of rs6505510 is associated with G alleles of both rs1889022 and rs10853181 on the same haplotype, and Salam *et al.* [25] reported that haplotypes carrying both G alleles (Major alleles) of rs1889022 and rs10853181 were associated with higher FeNO levels than non-carrying, consistent with our observations on the C allele of rs6505510.

Results regarding the associations of these genes with asthma are available from the European Gabriel consortium (http://www.cng.fr/gabriel/results.html), the largest GWAS of asthma conducted to date on more than 10,000 cases and 16,000 controls. No robust association between SNPs in the three *NOS* genes and asthma was found [26]. Further, none of the SNPs belonging to either of the three genes was reported associated with asthma or atopy in the Catalog of Published Genome-Wide Association Studies (http://www.genome.gov/gwastudies). To our knowledge, no partner of the Gabriel consortium has such complete data in adults as those investigated in the present paper, and no genetic study searching for associations between genetic variants belonging to *NOS* genes and NO_2_–NO_3_ levels in EBC has been published to date in the context of asthma or atopy. Further genetic studies are needed to confirm our findings including a possible differential effect of *NOS* variants on FeNO and EBC NO_2_–NO_3_ in subjects with and without asthma. We did not find any significant association between *NOS1* variants and any biological phenotype. This may be partly explained by the higher number of SNPs in that gene than in *NOS2* and *NOS3*, and thus the more stringent threshold used to correct for multiple testing. However, regarding FeNO levels, our results are consistent with recent observations in Swedish adults [24].

In conclusion, this study identified *NOS2* and *NOS3* polymorphisms associated with higher levels of EBC NO_2_–NO_3_ and Fe_NO_, and evidenced a modifying effect of asthma status on the associations between *NOS3* genetic variants and Fe_NO_ levels. Moreover, our findings highlight the critical relevance to have access to specific phenotypes measured in the relevant biological fluid (e.g. exhaled phenotypes). Genetic epidemiological studies of biological phenotypes involved in the same pathway can provide relevant information, and can contribute to disentangle the mechanisms underlying complex diseases such as asthma.

## Methods

### Study design

**Figure 1:**
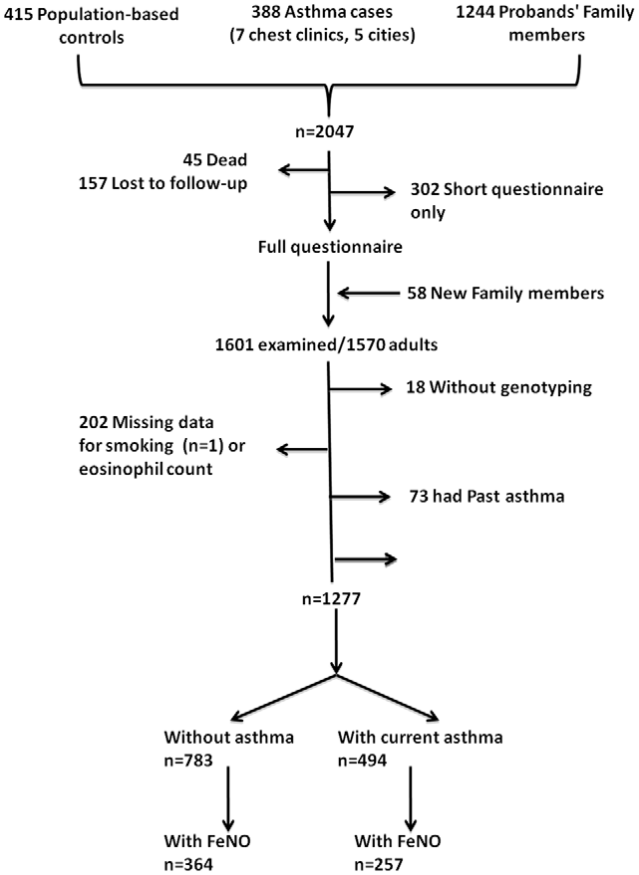
Flowchart of the studied population.

The Epidemiological study on the Genetics and Environment of Asthma (EGEA) combines a case-control study and a family study of asthmatic cases (http://cesp.vjf.inserm.fr/~egeanet/). The protocol and descriptive characteristics have been described elsewhere [27], [28]. Probands (asthmatic patients) were recruited from chest clinics between 1991 and 1995 and family members of asthmatic probands were included, either by including the proband's parents and siblings, or by including the proband's spouse and children. In addition, population-based controls were recruited. A follow-up of the initial cohort was conducted between 2003 and 2007 [29]. All subjects responded to a questionnaire based on international standardized tools to diagnose asthma and to determine respiratory and allergic symptoms, treatments, and environmental exposures [28]. The present cross-sectional analysis uses data from the follow-up in 1277 adult subjects with complete data on eosinophils, NO-related phenotypes, current asthma and genotypic data (Fig. 1). Ethical approval was obtained from the relevant institutional review board committees (Cochin Port-Royal Hospital and Necker-Enfants Malades Hospital, Paris). Written informed consent was signed by all participants. Written informed consent was signed by kin or guardians of the minors/children.

### Phenotypes

Inclusion criteria used to define asthma in probands were based on self-reported answers to the four questions: “Have you ever had attacks of breathlessness at rest with wheezing?”, “Have you ever had asthma attacks?”, “Was this diagnosis confirmed by a physician?”, and “Have you had an asthma attack in the last 12 months?”, or a positive response to at least two questions and a positive review of their medical record [29]. Asthma in relatives of probands was defined as a positive answer to at least one of the first two questions [29]. Among asthmatics, “current asthma” was defined by a report of respiratory symptoms in the past 12 months (wheeze, nocturnal chest tightness, attacks of breathlessness following strenuous activity, at rest or at night time, and asthma attacks) or use of inhaled and/or oral medicines because of breathing problems. Asthmatic subjects without current asthma were excluded from the present study. Lung function tests with methacholine challenge, total IgE and allergy skin prick tests were performed (see File S1).

### Biological phenotypes in the nitrate-nitrite-NO pathway

Exhaled breath condensate (EBC) was collected according to a standardized method using RTube™. Total nitrite-nitrate (NO_2_–NO_3_) levels were measured by the Griess reaction [30]. Briefly, nitrate in plasma or in EBC were reduced to nitrite by adding nitrate reductase (25 mU/ml) and NADPH 20 mM at room temperature and in dark. After 3 hours, samples were deproteinized by adding a solution of ZNSO4 30% and centrifuged. Griess reagent (0.1% naphthalethylene-dimine and 1% sulfanilamide in 5% H3PO4) was added to supernatants. The optical density at 560 nm was measured using a microplaque reader. Nitrate levels were calculated by comparison with optic density 560 of standard solutions of sodium nitrite. All measurements were done in duplicate. Analytical intra-run imprecision (CV) of these systems was below 3%. Measurements with coefficient of variation>15% and extreme outliers (n = 7) were excluded from the analyses. Protein concentration in EBC was determined according to Smith et al [31]. Total NO_2_–NO_3_ level was expressed as µM in plasma and as µmol/mg of proteins in EBC.

Fe_NO_ measurements at the 50 mL/s flow rate were realized according to ATS/ERS recommendations [21] before other pulmonary function tests as previously described [29]. Eosinophil count (EOS) was obtained from white blood cell count.

### Genotyping

We used all SNP data in *NOS1*, *NOS2* and *NOS3* available in the EGEA study to maximize the SNP density. These data included 18 SNPs genotyped using Taqman Probes (Applied Biosystems, Foster City, CA) on an ABI7900HT Sequence Detection System as part of a previous candidate gene project and 110 SNPs genotyped using the Illumina 610Quad array (Illumina, San Diego, CA) as part of the Gabriel Consortium GWAS (Table S1). The whole genotyping was performed at the Centre National de Génotypage (CNG, Evry, France). Consistency of the SNP data with Mendelian inheritance was evaluated using the PEDCHECK program [32]. Test of Hardy-Weinberg equilibrium was performed in non-asthmatic subjects using an exact test [33] (Table S1). After a quality control (QC) process, we selected 121 SNPs belonging to *NOS1* (77 SNPs), *NOS2* (37 SNPs) and *NOS3* (7 SNPs) and fulfilling the following QC criteria: call rate≥97%, minor allele frequency≥5%, and Hardy-Weinberg (HW) *P*-value>10^−4^. Pairwise linkage disequilibrium (LD) measures (r^2^) between polymorphisms of each of the three *NOS* genes were estimated using Haploview [34].

### Statistical Methods

Analyses were conducted on the four quantitative phenotypes under study (NO_2_–NO_3_ both in plasma and in EBC, Fe_NO_ and EOS) in subjects with and without asthma separately, because EOS count and Fe_NO_ are strongly associated with asthma, and because of the study design. We examined the log-transformed values of EOS and NO_2_–NO_3_, adjusted for age and sex and log-transformed Fe_NO_ adjusted for age, sex, height, smoking status (current, ex- and non-smokers) and centre. Association between each of these biological phenotypes and each of the 121 SNPs (single marker analysis) was investigated by linear regression using generalized estimated equations (GEE, MIXED procedure) to take into account the familial dependencies [35] and adjusting for principal components to capture population ancestry. The effect of each SNP (on each biological phenotype) was tested under three genetic models (additive, recessive and dominant) and the best-fitting model was selected. As FE_NO_ levels are highly influenced by inhaled corticosteroids (ICS), association analyses in asthmatic subjects were restricted to those who did not receive ICS in the last three months before the FE_NO_ measurement. For each phenotype under study, we removed outliers defined as samples beyond the mean±3 Standard Deviation units before any association analysis. The normality of phenotype distribution was tested using the Kolmogorov-Smirnov (K–S) test in asthmatic and non-asthmatics separately, and only two tests were marginally significant.

To correct for the effect of testing multiple SNPs within each gene, we estimated the effective number of independent SNPs (Meff) using Li and Ji's method [36], which is based on the pairwise LD measure, r^2^, between SNPs. This method has the advantage to take into account the linkage disequilibrium (LD) between SNPs under study, and provides an accurate approximation to the permutation based correction threshold [36]. A Bonferroni correction was then applied using the Meff estimates leading to p-value thresholds of 0.0015, 0.0024 and 0.01 for *NOS1*, *NOS2* and *NOS3* respectively. We did not further correct for the number of biological phenotypes and for the number of genes as the four phenotypes and the three genes are not independent. For the significant SNPs, we formally tested for heterogeneity of each SNP effect on the phenotype between non-asthmatic and asthmatic subjects, under the best fitting genetic model, by introducing a SNP x asthma interaction term in the regression model. In order to detect independent effects of SNPs belonging to a gene, we performed stepwise regression by examining within each gene all SNPs with a p-value less than 0.10 in single marker-analysis and with pairwise LD r^2^<0.80. All statistical analyses were done using SAS version 9.2 (SAS Institute, Inc., Cary, NC). Statistical power was computed using Quanto 1.2.4 (University of Southern California, USA, http://hydra.usc.edu/gxe).

## Supporting Information

File S1 Supplemental data regarding methods (phenotypes and genotyping).
(DOC)

Table S1 SNP identification number, genotyping method, position, minor allele frequency (MAF) and test of Hardy-Weinberg (HW) equilibrium (P value) for 121 SNPs belonging to *NOS1*, *NOS2* and *NOS3* genes.
(XLS)

Table S2 Associations between Single Nucleotide Polymorphisms belonging to *NOS1*, *NOS2* and *NOS3* with Fe_NO_ level according to asthma status (univariate analyses).
(XLS)

Table S3 Associations between Single Nucleotide Polymorphisms belonging to *NOS1*, *NOS2* and *NOS3* with total NO_2_–NO_3_ level in exhaled breath condensate according to asthma status (univariate analyses).
(XLS)

Table S4 Associations between Single Nucleotide Polymorphisms belonging to *NOS1*, *NOS2* and *NOS3* with total NO_2_–NO_3_ level in plasma according to asthma status (univariate analyses).
(XLS)

Table S5 Associations between Single Nucleotide Polymorphisms belonging to *NOS1*, *NOS2* and *NOS3* with blood eosinophil count according to asthma status (univariate analyses).
(XLS)
