# Supplementary material for: Associations between Nitric Oxide Synthase Genes and Exhaled NO-Related Phenotypes according to Asthma Status
Source: PLoS One. 2012 May 9;7(5):e36672. doi: 10.1371/journal.pone.0036672 (PMC3348876; doi:10.1371/journal.pone.0036672)
Supplement: File S1 — Supplemental data regarding methods (phenotypes and genotyping). (DOC) [file pone.0036672.s001.doc]

SUPPLEMENTAL DATA

**Associations between Nitric Oxide Synthase Genes and Exhaled NO-related Phenotypes according to Asthma Status**

Emmanuelle Bouzigon^1,2,3^, Florent Monier^4,5^, Mekki Boussaha^1,2,3,#^, Nicole Le Moual^4,5^, Hélène Huyvaert^6,7^, Régis Matran^6,7^, Sébastien Letort^1,2,3^, Jean Bousquet^4,8^, Isabelle Pin^9,10,11^, Mark Lathrop^12^, Francine Kauffmann^4,5^, Florence Demenais^1,2,3^, Rachel Nadif^4,5^, on behalf of the EGEA Cooperative Group

The EGEA Cooperative group is a group authorship.

# METHODS

***Phenotypes***

Baseline spirometry was performed using a standardized protocol with similar equipment across centres according to the ATS/ERS guidelines. [1]. Methacholine challenge was performed unless baseline FEV1 <80% predicted.

Skin Prick Test positivity (SPT+) was defined by a mean wheal diameter ≥3mm than the negative control for at least one of 11 aeroallergens (cat, *Dermatophagoides pteronyssinus*, *Blattela germanica*, olive, birch, *Parieteria judaica*, timothy grass, ragweed pollen, *Aspergillus*, *Cladosporium herbarum*, *Alternaria tenuis*). A quantitative score was constructed as the number of positive test results (SPTQ) and validated regarding its biometric properties [2].

***Genotyping***

We used all genotyping data belonging to *NOS1*, *NOS2* and *NOS3* available in EGEA study. The genotyping was performed either using Taqman Probes (Applied Biosystems, Foster City, CA) on an ABI7900HT Sequence Detection System as part of a previous candidate gene project or using the Illumina 610Quad array (Illumina, San Diego, CA) as part of the Gabriel Consortium (18 SNPs and 110 SNPs respectively). All genotyping were performed at the Centre National de Génotypage (CNG, Evry, France). Consistency of the SNP data with Mendelian inheritance was evaluated using the PEDCHECK program [3]. Test of Hardy-Weinberg equilibrium was performed using an exact test [4]. After a quality control (QC) process, we selected 121 SNPs belonging to *NOS1* (77 SNPs), *NOS2* (37 SNPs) and *NOS3* (7 SNPs) and fulfilling the following QC criteria: call rate ≥ 97%, minor allele frequency ≥ 5%, and Hardy-Weinberg (HW) *P*-value >10^-4^. Pairwise linkage disequilibrium (LD) measures (r^2^) between polymorphisms of each of the three *NOS* genes were estimated using Haploview [5].

**REFERENCES**

1. Miller MR, Hankinson J, Brusasco V, Burgos F, Casaburi R et al. (2005) ATS/ERS Task Force. Standardisation of spirometry. Eur Respir J 26: 319-338.

2. Maccario J, Oryszczyn MP, Charpin D, Kauffmann F (2003) Methodologic aspects of the quantification of skin prick test responses: the EGEA study. J Allergy Clin Immunol 111: 750-756.

3. O'Connell J, Weeks DE (1998) PedCheck: A Program for Identification of Genotype Incompatibilities in Linkage Analysis. Am J Hum Genet 63: 259-266.

4. Abecasis GR, Wigginton JE (2005) Handling marker-marker linkage disequilibrium: pedigree analysis with clustered markers. Am J Hum Genet 77: 754-767.

5. Barrett JC, Fry B, Maller J, Daly MJ (2005) Haploview: analysis and visualization of LD and haplotype maps. Bioinformatics 21: 263-265.
